# An effective snakebite first aid training method for medics in the Chinese troops: a RCT

**DOI:** 10.1186/s40779-019-0230-9

**Published:** 2019-12-13

**Authors:** Chen Qiu, Xiao-Feng Qiu, Jing-Jing Liu, Yi-Xin Wang, Li Gui

**Affiliations:** 1 First Medical Center, General Hospital of the PLA, Beijing, 100853 China; 2 Department of Emergency Nursing, School of Nursing, Naval Medical University, Shanghai, 200433 China; 3 Second Medical Center, General Hospital of the PLA, Beijing, 100853 China

**Keywords:** Snakebite, Standard operation procedure (SOP), Checklist, Medic, Training

## Abstract

**Background:**

Snakebites can lead to lifelong consequences and is one of the main causes of death among military troops worldwide. However, few Chinese military medics know the proper first aid procedures for snakebites. Therefore, this study aimed to explore the impact of the Standard Operation Procedure (SOP) and checklist on Chinese military medics’ ability to manage snakebite first aid.

**Methods:**

This study was a prospective single-blind randomized controlled trial conducted in a military medical university of China from May to June 2017. A questionnaire-based survey was performed to collect the participants’ socio-demographic profiles before the baseline measurement. During the baseline measurement, participants were requested to provide corresponding first aid that was responsive to the simulative situation portrayed by the SPs (standardized patients) and the evaluators then scored their performances according to a checklist for snakebite first aid scoring table. After the baseline measurement, they were randomly assigned to one of three intervention groups after stratification according to their baseline performance scores: group A received a self-learning course with textbooks (*n* = 27), group B received a self-learning training on the SOP and checklist (*n* = 27) and group C was engaged in an interactive discussion panel regarding the SOP and checklist (*n* = 26). After the interventions, participants received outcome measurements about snakebite first aid key points capability from the same evaluator and SP for each group to avoid observational error. The reviewers were blinded about the grouping in the trial.

**Results:**

The baseline measurement yielded no significant difference (*H* = 1.647, *P* = 0.439) among the three groups. The post-intervention scores were higher than the pre-intervention scores for all three (A, B and C) groups (*P* = 0.008, *P* < 0.001 and *P* < 0.001, respectively). There was significant difference of the post-intervention scores among the three groups (*F* = 8.841, *P* < 0.001). Both post-intervention scores of group B and group C were higher than that of group A (*P* < 0.001 and *P* = 0.001, respectively), but no difference was found between group B and C (*P* = 0.695). The acceptance questionnaire score of SOP and checklist was mostly very satisfied, as the final scores of group B and group C were 4.62 ± 0.61 and 4.82 ± 0.45, respectively.

**Conclusions:**

In this study, the implementation of an SOP and checklist for snakebite first aid was shown to update and improve first aid treatment concepts in military medics. These intervention methods played an important role in improving the medics’ cognition and understanding of snakebite first aid. Therefore, this finding suggests that SOP and checklist training should be further implemented in Chinese troops for snakebite care.

## Background

Snakebite is a persistent threat that can be acute and life-threatening and may lead to significant morbidity or long-term injuries among military troops who join in battles in the most inhospitable environments, such as jungles and hills, or are involved with other military duties. Approximately 2.7 million cases of snakebite have been reported worldwide each year [1], of which 20,000 are casualties. Annually, more than 100,000 military and civilian personnel in Afghanistan confront venomous snakes at least once [2]. Additionally, in many countries of Southeast Asia, snakebite is an important medical emergency and cause of hospital admission. In China, the mountains and jungles of most tropical and subtropical areas contain life-threatening snakes, which accounts for the high morbidity and mortality rate from snakebites. Thus, a snakebite demands urgent attention from adequately trained medical staff [3], specifically the first rescuer, who must know how to conduct first aid for snakebite. For this reason, the World Health Organization (WHO) recommends that education and training in the prevention and management of snakebites should be included in the curriculum of medical and nursing schools [3]. It is also of great importance to implement the training of medics in the grass-roots army.

The Standard Operation Procedure (SOP) and checklist are training methods that provide standards for medical education and help users quickly identify and correctly treat snakebite symptoms, thus resulting in significantly reduced medical errors and risks [4]. Furthermore, a flexible combination of the SOP and checklist is a high-quality approach to improve training efficiency [5]. Other similar methods have been widely used in education, specifically in medicine, such as the Advanced Cardiovascular Life Support (ACLS) course developed by the American Heart Association (AHA) [6], which has been used mainly in continuing education and training with the aim of assisting students to quickly refresh their memory and master their skills.

Considering this evidence, we developed an SOP and checklist for snakebite first aid guidance based on the Experiential Learning Theory (ELT) theoretical learning-centered rationale, which focuses on the overall analysis and emphasizes the process of learning, including the cognitive, environmental and emotional factors that influence it. The purpose of our research was to verify whether the designed SOP and checklist for snakebite first aid was meaningful and valuable as a training approach and if, consequently, it was able to improve medical knowledge, thus impacting the decision of whether to implement these tools in the future.

## Materials and methods

### Participants

This study was a prospective single-blind randomized controlled trial (RCT) conducted in a military medical university of China from May to June 2017. This study received ethical approval from the Institutional Review Board of the Naval Medical University (CHEC2015–037), and written informed consent was obtained from all participants. All volunteer participants in this study were military medics who were currently working in primary military hospitals and/or participating in professional medical training from a military medical university in China. Most of them joined the army or started service after high school graduation. The study also enrolled three reviewers to score the participants and three postgraduate students to act as standardized patients (SPs) who were bitten by snake. These reviewers were teachers with a strong background in emergency nursing training and blinded to the grouping information. Participants used an anonymous and self-paced method to answer the acceptance questionnaire. Only two researchers reviewed and categorized the completed data collection forms.

### Study preparation

Before the study, three evaluators and three SPs were invited to participate in a two-hour training session. Evaluators were previously trained on their different roles in preparation for the subsequent intervention steps. They each scored the participants by using the same snakebite first aid scoring table (see Part 1 of Additional file 1), and as a result, a final score was summed from the preliminary experiments. The SPs were also trained in snakebite field-simulation scenarios according to standardized cases, including fatalities, in order to facilitate their portrayal of a real-life snakebite incident as a patient (see Part 2 of Additional file 1).

### Sampling and grouping

After the baseline measurement, the scores of each participant were sorted in a descending order and were divided into three tertiles: the top third, the middle third and the last third. A stratified randomization was conducted in each tertiles to separate the participants into three groups (group A, B and C) for intervention.

### Measurement tools and baseline measurement

Two weeks before the baseline measurements, all participants were invited to fill out a self-designed socio-demographic questionnaire (see Part 3 of Additional file 1) that was used to collect socio-demographic information, including gender, age, years of service in the army, years spent working in the medical industry, education level, specialty and work units. After that, participants were invited to take a 30-min course on snakebite first-aid to avoid a ‘blank questionnaire’ that might influence the final analysis. During baseline measurement, participants were requested to provide corresponding first aid that was responsive to the simulative situation portrayed by the SPs, and the evaluator then scored their performances. The snakebite field-simulation scenarios were elaborated according to the literature of field medicine and input from experts in field treatment. Random number table was utilized to decide which team of evaluators and SPs evaluate each participating medics for their ability of snakebite first aid. The scoring table combined with a checklist for snakebite first aid was formulated, and both the SOP acceptance questionnaire and checklist (see Part 4 of Additional file 1) were developed according to the 5-point Likert scale, on which participants scored their satisfaction from 1 to 5 points (from 1 point = very dissatisfied progressing to 5 points = very satisfied). All measurements met reliability and accuracy criteria in accordance with experts’ previous revision.

### Interventions

Two weeks after the baseline measurements and the following grouping strategy, interventions were conducted to check for any improvements. Three intervention methods were designed as follows: group A (*n* = 27) took a self-learning course with textbooks, which is one of the most common approaches for medics to acquire knowledge; group B (*n* = 27) took a self-learning course with our proposed SOP combined with checklist, and no intra-group communication was allowed; and group C (*n* = 26) was engaged in a proactive discussion panel about the SOP and checklist. In group C, we encouraged communication and discussion among group members. Each group was conducted simultaneously, and the interventions lasted an hour. All participants were forbidden to contact each other during the test to avoid group feedback or interference in the testing process.

### Post-intervention measurements

After the interventions, participants received outcome measurements about snakebite first aid capability from the same evaluator and SP for each group to avoid observational error. The post-intervention measurement procedure was the same as described for the baseline measurement and was performed immediately after the intervention to evaluate the impact of the interventions in groups A / B/ C. To avoid repetitive evaluation errors, equivalent SP cases with different backgrounds were conducted. Finally, participants in Groups B and C were requested to complete, by themselves and anonymously, an SOP acceptance questionnaire and checklist lasting 15 min at maximum.

### Data collection and statistical analysis

Participants were awarded with one positive point for each correct response and one point was subtracted for each incorrect response. No points were awarded for blanked-responses. The summation of all the response scores equaled the final score. For statistical analyses, Microsoft Excel 2010 (Microsoft Corp, USA) and SPSS 20.0 (IBM Corp, USA) software were used to collect and analyze the data. Continuous variables were reported as the means±standard deviation, while categorical variables were represented as counts and percentages of the respondents to the overall participants. The difference of each comparison was considered to be significant when *P* was reported to be less than 0.05. The analysis of variance (ANOVA) test was used to compare between-group differences as well as baseline differences in variables with a normal distribution. The Kruskal-Wallis *H* test was used to analyze data with a non-normal distribution. The Newman Keuls test was applied after ANOVA when appropriate. The *Z*-test was used to compare the different rates of responses between the groups as well as pre- and post-intervention within each group. We calibrated α = 0.05/3 = 0.0167, therefore *P* < 0.0167 was considered statistically significant.

## Results

### Socio-demographic information and baseline assessment of the participants

Out of the 89 medics invited to participate in the study, only 80 medics were able to follow-up with the study and be considered for the statistical analysis while the rest 9 medics dropped out due to duty, illness and other reasons. The average age of the participants was 24.6 ± 1.98 years old, and their average working years in the medical field was 5.2 ± 2.0 years. Table 1 shows these and other relevant socio-demographic information. No significant differences were found among the A, B, and C groups (Table 1).

**Table 1:** Socio-demographic information of the participants [*n*(%)])

Characteristics	Group A	Group B	Group C	*χ*^2^/*F*	*P*
Gender					
Male	22(27.5)	25(31.3)	23(28.8)	1.56	0.46
Female	5(6.3)	2(2.5)	3(3.8)		
Age (year)					
20–24	14(17.5)	12(15)	9(11.3)	1.61	0.45
25–29	13(16.3)	15(18.8)	17(21.3)		
Education level					
High school degree	11(13.8)	10(12.5)	12(15)	1.69	0.79
Junior college degree	15(18.8)	17(21.25)	13(16.3)		
Bachelor’s degree	1(1.25)	0(0)	1(1.3)		
Working years in the medical field					
1–3	6(7.5)	7(8.75)	6(7.5)	1.60	0.81
4–7	16(20)	17(21.2)	18(22.5)		
8–10	5(6.3)	3(3.8)	2(2.5)		
Have ever dealt with snakebite?					
Yes	7(8.8)	5(6.3)	7(8.8)	0.62	0.73
No	20(25)	22(27.5)	19(23.7)		

### Overall group differences between baseline and post-intervention measurements

From the 80 enrolled participants in this single-blind randomized controlled trial, participants were randomly distributed to group A (*n* = 27), group B (*n* = 27) and group C (*n* = 26). To verify whether the training approaches for snakebite first aid consisting of an SOP and checklist were meaningful for a military medical setting, we first compared scores in both intra- and inter-group after the different educational intervention approaches. We found that the post-intervention measurement was higher than the pre-intervention measurement for all three A, B and C groups (*P* = 0.008, *P* < 0.001 and *P* < 0.001, respectively, Table 2) and that this difference was significant among the three groups (*F* = 8.841, *P* < 0.001). Further analysis showed that the post-intervention scores of both group B and group C were higher than that of group A (*P* < 0.001 and *P* = 0.001, respectively), but no difference was found between group B and C (*P* = 0.695, Table 2).

**Table 2:** Inter-group and intra-group comparison of the baseline and post-intervention scores (*n* = 80)

Group	Baseline scores	Post-intervention scores	*t* value	*P*
A	61.2 ± 14.0	70 ± 9.1	2.89	0.008
B	58.3 ± 10.8	87.2 ± 7.8^(1)^	7.078	< 0.001
C	60.1 ± 14.2	86.8 ± 7.1^(2)^	6.71	< 0.001
*H*/*F*	1.647^(H)^	8.841		
*P*	0.439	< 0.001		

Compared with group A, (1) *P*<0.001, (2) *P* = 0.001. (H) indicates that a Kruskal-Wallis test was performed. When comparing post-intervention scores with baseline scores, *P*<0.05 was considered as statistical significant.

### Improvements in answers to snakebite first aid key-points after the educational intervention

To further analyze the participants’ snakebite first aid performance improvements after the intervention, we comprehensively analyzed key-point answers provided by all three groups the pre- and post-intervention measurements, as shown in Table 3. Our results showed that, before the intervention (baseline), 62 out of 80 participants chose one or more wrong measures in snakebite first aid, such as tourniquet ligation, cupping, sucking or squeezing the venom out, wound incision, or alcohol/iodophor disinfection. Moreover, only 15 (18.75%) participants used the pressure immobilization technique, and although 6 (7.5%) participants injected epinephrine for patients with allergic shock, only one (1.25%) participant decided to perform re-injection of epinephrine 5 min after ineffectiveness of the first injection.

**Table 3:** Comparison of key points from the scoring table between baseline and post-intervention measurements [*n* (%)]

Key points	Group A (*n* = 27)					Group B (*n* = 27)			Group C (*n* = 26)					Group A vs B	Group A vs C		Group B vs C	
	Baseline	Post-intervention	Rate difference	*P*	Baseline	Post-intervention	Rate difference	*P*	Baseline	Post-intervention	Rate difference	*P*	post-intervention rate difference	*P*	post-intervention rate difference	*P*	post-intervention rate difference	*P*
Right measures																		
Rinse wound instantly	12 (44.4)	18 (66.7)	0.22	0.05	12 (44.4)	25 (92.6)	0.48	< 0.01	15 (57.7)	22 (84.6)	0.27	0.01	0.26	0.01	0.18	0.07	0.08	0.18
Oral or percutaneous snake drug administration	25 (92.6)	25 (92.6)			22 (81.5)	22 (81.5)			24 (92.3)	21 (80.8)								
Keep stationary	5 (18.5)	17 (63)	0.44	< 0.01	5 (18.5)	15 (55.6)	0.37	< 0.01	4 (15.4)	18 (66.7)	0.50	< 0.01	0.07	0.29	0.02	0.43	0.10	0.23
Pressure immobilization technique	8 (29.6)	16 (59.3)	0.30	0.01	5 (18.5)	17 (63)	0.44	< 0.01	2 (7.7)	17 (65.4)	0.58	< 0.01	0.04	0.39	0.06	0.32	0.02	0.43
Stretcher transport	11 (40.7)	9 (33.3)			9 (33.3)	18 (66.7)			13 (50)	21 (80.8)								
Assess patient’s condition and monitor the complications	5 (18.5)	2 (7.4)			5 (18.5)	3 (11.1)			6 (23.1)	5 (19.2)								
Diagnosis of allergic shock	8 (29.6)	4 (14.8)			8 (29.6)	5 (18.5)			5 (19.2)	12 (46.2)								
Obtain IV access	7 (25.9)	7 (25.9)			5 (18.5)	17 (63)			6 (23.0)	19 (73.1)								
0.5 mg IM/H epinephrine	3 (11.1)	10 (37)	0.30	0.01	2 (7.4)	17 (63)	0.56	< 0.01	1 (3.9)	17 (65.4)	0.62	< 0.01	0.26	0.03	0.28	0.02	0.02	0.43
10 mg IV dexamethasone	8 (29.6)	12 (44.4)			8 (29.6)	16 (59.3)			10 (38.5)	15 (57.7)								
GTT saline IV for expansion	4 (14.8)	1 (3.7)			2 (7.4)	8 (29.6)			5 (19.2)	6 (23.1)								
If ineffective in 5 min, repeat injection of epinephrine	1 (3.7)	4 (14.8)	0.11	0.08	0	14 (51.9)	0.56	< 0.01	0	15 (57.7)	0.58	< 0.01	0.37	< 0.01	0.43	< 0.01	0.06	0.34
Evacuation	18 (66.7)	16 (59.3)			19 (70.4)	25 (92.6)			18 (66.7)	25 (96.2)								
Wrong measures																		
Tourniquet ligation	22 (81.5)	8 (29.6)	0.11	0.08	19 (70.4)	3 (11.1)	0.59	< 0.01	17 (65.4)	5 (19.2)	0.46	< 0.01						
Cupping therapy	5 (18.5)	1 (3.7)	0.15	0.04	5 (18.5)	0	0.19	0.01	5 (19.2)	0	0.19	0.01						
Suck, squeeze venom out	3 (11.1)	3 (11.1)	0.00	0.5	3 (11.1)	1 (3.7)	0.07	0.15	1 (3.9)	0	0.04	0.16						
Incise the wound	13 (48.2)	6 (22.2)	0.26	0.02	12 (44.4)	1 (3.7)	0.41	< 0.01	8 (30.8)	1 (3.9)	0.27	0.01	0.19	0.02	0.18	0.02	0.00	0.49
Alcohol/iodophor disinfection	2 (7.4)	1 (3.7)			1 (3.7)	0			3 (11.5)	0								
Noradrenaline/norepinephrine	1 (3.7)	0			0	0			0	1 (3.9)								
Chlorphenamine maleate	5 (18.5)	1 (3.7)			4 (14.8)	0			4 (15.4)	0								

Statistical analysis was performed in comparing the core key points, and the blanked cells in the table represent non-core key points.

In contrast, a reduced number of participants still chose the wrong methods measures for snakebite first aid after the educational intervention (post-intervention measurement). Of those who indicated the wrong methods, 10 belonged to group A, 3 to group B and 5 to group C. Among all participants, 50 (62.5%) immediately applied the pressure immobilization technique, and the number of participants who selected injecting epinephrine for allergic shock also improved from baseline, with a total of 44 participants (10 in group A, 17 in G group B and 17 in group C) choosing this method after the intervention. Additionally, we verified that, in the post-intervention measurements, 33 participants (4 from group A, 14 from Group B, and 15 from Group C), in contrast to only one participant in the pre-intervention, decided to perform the re-injection of epinephrine 5 min when the first injection turned out to be ineffective.

### Intra- and inter-group differences in regard to answers for key points between baseline and post-intervention measurements

In regard to inter-group differences between baseline and post-intervention measurement, we found no statistically significant differences in the key-point answers to “keep stationary” and “pressure immobilization technique” (*P* > 0.0167) items, in contrast to “IM/H 0.5 mg epinephrine” and “cupping therapy”, which were answered significantly different between participants from group A vs B and group A vs C, respectively (*P* < 0.0167).

### SOP and checklist acceptance questionnaire

We implemented the SOP and checklist acceptance questionnaire to check participants’ satisfaction with the training provided by this study and whether they considered it relevant for their medical treatment skills for snakebite first aid. In this regard, we found that the acceptance score was mostly very satisfied, as the final scores of group B and group C were 4.62 ± 0.61 and 4.82 ± 0.45, respectively. Even though the key-point item “met my learning needs” received the lowest mean score of 4.37 ± 0.97 in group B, the highest mean scores of 4.74 ± 0.45 and 4.88 ± 0.33 were achieved in both groups B and C, respectively, with the item: “effectively updated my concept of treatment”. Overall, the data showed that the scores for all items in group C were higher than those in group B, and the specific items “met my learning needs”, “stimulated my interest in learning”, “easy to comprehend” were significantly different between groups B and C (*P* < 0.05). The statistical results of the SOP and checklist acceptance questionnaire are listed in Table 4.

**Table 4:** Questionnaire assessing the acceptance of SOP and checklist (mean ± standard deviation)

Items	Group B	Group C	*U*	*P*
Met my learning needs	4.37 ± 0.97	4.85 ± 0.46	237.50	0.01
Effectively updated my concept of treatment	4.74 ± 0.45	4.88 ± 0.33	300.50	0.19
Stimulated my interest in learning	4.44 ± 0.70	4.81 ± 0.63	238.50	0.01
Integrated theory with practice	4.67 ± 0.55	4.77 ± 0.51	316.00	0.41
Easy to master	4.67 ± 0.55	4.77 ± 0.51	316.00	0.41
Guided me to effective treatment	4.70 ± 0.47	4.81 ± 0.40	314.50	0.38
Improved my self-confidence on treatment	4.70 ± 0.47	4.81 ± 0.40	314.50	0.38
Enhanced decision-making ability	4.67 ± 0.48	4.85 ± 0.37	288.00	0.13
Assisted me in enhancing my sense of self-identity	4.63 ± 0.69	4.81 ± 0.40	312.00	0.35
Comprehensive effects	4.62 ± 0.61	4.82 ± 0.45	246.00	0.03

## Discussion

Snakebites are environmental, occupational and climatic hazards, especially in the field of military operations. Actually, snakebites are considered a major disease in military personnel by the WHO [3], as they may lead to a considerable number of deaths or chronic disability each year in in this population if the treatment is delayed or incorrect. However, little attention has been paid to the true scale of mortality as well as to the acute and chronic morbidities as a consequence of snakebite, as shown in a community-based study [7]. To aggravate this condition, prevention and first-aid training for snakebites have not been recognized by the Chinese army. As the main force of military primary health care, medics take on all of the nursing responsibilities in a military unit on or below the regimental level [8]. However, as shown in this study, the result of the baseline measurements demonstrated that the medics’ cognition and snakebite first-aid abilities were diminished and with potential procedure errors. Under these circumstances, developing a systematic and comprehensive training method for snakebite first-aid is urgently needed. In addition, strengthening medical education, as well as the propaganda around snakebites, is also important.

A previous study has shown that the implementation of an SOP and checklist can significantly decrease the error rate [9]. Based on that, we have chosen to implement an SOP and checklist in this study to improve medics’ snakebite first-aid knowledge. For this, we adopted three kinds of independent interventions, which comprised self-learning on the basis of textbooks (group A), self-learning guided by the SOP and checklist (group B) and a panel discussion in addition to the SOP and checklist (group C). We observed that when compared to group A, the error rate for key-point answers from participants in both groups B and C were lower, thus indicating that medical error risk was reduced for those groups. For instance, trainees in groups B and C, who were trained in the SOP and checklist learning methods, rarely selected incorrect measures for dealing with snakebite first-aid, such as “incising the wound”, and “sucking or squeezing the venom out”.

Based on these results, we observed that although all trainees, even the ones that received the self-learning solely with textbook support and without the help of the SOP and checklist, gained a certain level of improvement on snakebite first-aid. Furthermore, there was a superior performance of medics who were trained with the SOP and checklist, as they could better identify symptoms and chose the correct interventions. Based on these results, we demonstrated that the interventions in this study can significantly impact and improve the participants’ cognition and knowledge of snakebite first-aid and diminish error rates for key-point procedures as much as possible after a short period of 2 weeks from baseline. We believe that this result may relate to the fact that snakebite injuries can be accessible after SOP training. However, the effectiveness and the final performance in the post-intervention measurements varied among the three intervention groups and may be related to many factors.

First, as previously reported, the SOP and checklist have been shown to be useful in preventing errors and reducing morbidity and mortality [9]. Additionally, in accordance with this report, the use of the SOP and checklist by groups B and C resulted in a lower ratio of key-point mistakes and, consequently, a lower risk of first-aid errors than in group A. Therefore, the SOP and checklist helped participants better improve their knowledge and skills compared with those only had access to self-learning on the basis of textbooks. What’s more, there were fewer participants in groups B and C who selected the wrong procedures, such as “tourniquet ligation”, “cupping therapy”, “sucking or squeezing the venom out”, “wound incision”, or “alcohol/iodophor disinfection”. The above procedures have been shown to worsen patients’ state of illness [10, 11].

Second, with the help of the SOP and checklist, medics identified the symptom correctly so that they were able to choose the right treatment. For the snakebite field-simulation case of a patient with allergic shock, the number of correct answers regarding epinephrine dosing in groups B and C was 6.33-fold higher in the post-intervention measurements than in the pretest measurement and 33-fold higher among the participants who had originally chosen to reinject epinephrine in patients 5 min after the ineffective first injection in comparison to group A. This observation can be attributed to the concise list of key steps in the SOP and checklist. Other studies, such as the study conducted by Katja et al. [12], also tested the effectiveness of checklists for emergency procedures on medics’ performance in intensive care unit (ICU) crises and demonstrated the benefit of using a checklist in this scenario to ensure the completion of critical treatment steps.

Third, the results from the SOP acceptance questionnaire showed a high acceptance of the intervention methods. The general acceptance in group C was higher than in group B, which indicated that the panel discussion had a significant impact. Moreover, the adoption of the SOP and checklist not only improved the medics’ current knowledge levels but also decreased the dependence of the medic on surgeons as the SOP and checklist contributed to the medics’ independent decision-making, self-acceptance and self-confidence, which is a drawback for treatment operations in the army [13]. This can be explained by the fact that medics are in great demand for field-based first aid knowledge, and in this matter, the SOP and checklist were helpful to improve the medics’ abilities. Further professional training or relevant study materials are also necessary for future improvement.

Despite these favorable results, the main limitation of this study is that the scenarios have been tested in a limited sample size and study results can be more persuasive if larger populations were studied. In this matter, we are aware that, before adopting the SOP and checklist for use in standardized casualties and cases, we must first ensure that they have been validated with fidelity and effectiveness in field simulations or other approaches involving a larger number of participants, thus providing a more robust estimate of effect. Some other points that can be further implemented included the improvement of the timing period during the participants’ training sessions by providing them more time to learn, which may result in enhanced differences in the learning process, and the implementation of a third test to evaluate if acquired knowledge from training is long-lasting.

Finally, we concluded in this study that supplementing traditional textbook-based education with an SOP and checklist is able to comprehensively improve medics’ field first-aid knowledge. In this regard, we recommend this method as a preferable choice to train a wide range of medics in first-aid protocols as it is more feasible and accessible to conduct the SOP and checklist training method than to implement a complex traditional training method. Therefore, the SOP and checklist training method is a good supplement to the traditional training method for a wide range of military medics. It is also suggested to enlarge the sample volume and to maintain newer versions of SOP and checklist for updated practices. Additionally, we highlight that the health department in the army should be equipped with more necessary emergency equipment and supplies, such as antidotes for snakebites. Furthermore, in terms of preventive measures, soldiers in risky areas should wear personal protective equipment (PPE), which effectively guards against all jellyfish stings and snakebites, as it is routinely recommended for every army soldier in Australia [14].

## Conclusions

Basic skills for snakebite on-site identification and first aid procedures are inadequate in medical Chinese army troops. This study had the purpose of measuring the improvements of medics’ on-site snakebite first aid abilities after an SOP and checklist training based on self-learning and a panel discussion. Our outcomes suggested that the training methods implemented here can improve the identification of symptoms and the choice for correct interventions by medics. Therefore, further efforts should be made to make the SOP and checklist widely accessible for military troops.

## Additional file

**Additional file 1.** Part 1. Scoring table for snakebite first aid. Part 2. Snakebite scenarios. Part 3. A socio-demographic information questionnaire. Part 4. Acceptance questionnaire of the SOP and checklist.

## Data Availability

The supporting data are available.
