# Supplementary material for: An effective snakebite first aid training method for medics in the Chinese troops: a RCT
Source: Mil Med Res. 2019 Dec 13;6:39. doi: 10.1186/s40779-019-0230-9 (PMC6909628; doi:10.1186/s40779-019-0230-9)
Supplement: Supplementary file 1 — Additional file 1. Part 1. Scoring table for snakebite first aid. Part 2. Snakebite scenarios. Part 3. A socio-demographic information questionnaire. Part 4. Acceptance questionnaire of the SOP and checklist. [file 40779_2019_230_MOESM1_ESM.docx]

**Supplementary Materials**

**Part 1. Scoring table for snakebite first aid**

| **No.** | **Key point from checklist** |  | **Respondent 1** | **Respondent 2** |  | **Respondent 3** |  |
| --- | --- | --- | --- | --- | --- | --- | --- |
| B.1. | Rinse wound instantly |  |  |  |  |  |  |
| B.2. | Take the snake drugs orally or apply externally |  |  |  |  |  |  |
| B.3. | Keep stationary |  |  |  |  |  |  |
| B.4. | Pressure immobilization technique |  |  |  |  |  |  |
| B.5. | Stretcher transport |  |  |  |  |  |  |
| B.6. | Assess patient’s condition and monitor the complications |  |  |  |  |  |  |
| B.7. | Diagnosis of allergic shock |  |  |  |  |  |  |
| B.8. | Prostrate |  |  |  |  |  |  |
| B.9. | Keep breathing smoothly |  |  |  |  |  |  |
| B.10. | Oxygen therapy |  |  |  |  |  |  |
| B.11. | Obtain IV access |  |  |  |  |  |  |
| B.12. | 0.5 ml IM/H epinephrine |  |  |  |  |  |  |
| B.13. | 10 mg IV dexamethasone |  |  |  |  |  |  |
| B.14. | GTT saline IV for expansion |  |  |  |  |  |  |
| B.15. | If no improvement in five minutes, repeat injection of epinephrine |  |  |  |  |  |  |
| B.16. | Evacuation |  |  |  |  |  |  |
|  | **Wrong measures** |  |  |  |  |  |  |
| B.17. | Tourniquet ligation |  |  |  |  |  |  |
| B.18. | Cupping therapy |  |  |  |  |  |  |
| B.19. | Suck, squeeze venom out |  |  |  |  |  |  |
| B.20. | Incise the wound |  |  |  |  |  |  |
| B.21. | Alcohol/iodophor disinfection |  |  |  |  |  |  |
| B.22. | Noradrenaline/norepinephrine |  |  |  |  |  |  |
| B.23. | Chlorphenamine maleate |  |  |  |  |  |  |

**Part 2. Snakebite scenarios**

***Case 1. Snakebite scenario for pre-intervention***

A 22-year-old male soldier’s right leg was bitten accidentally by an unknown species of snake during the wilderness survival training. The patient was found conscious at the accident scene with a painful expression and wound swelling.

**Condition evolution:** The patient required activities after bandaging, with skin itching and bronchial asthma following.

**Examiner Tip:** given epinephrine if ineffective

**If you are in the field, how would you deal with this case?**

***Case 2. Snakebite scenario for post-intervention***

A 29-year-old male military cadet’s left leg was bitten accidentally by an unknown species of snake while conducting a topographic examination in the mountain. The patient was found conscious at the accident scene with painful expression and wound swelling.

**Condition evolution:** Asthma, dyspnea while in the observation room. (That occurs during anaphylactic shock)

**Examiner Tip:** given epinephrine if ineffective

**If you are in the field, how would you deal with this case?**

**Part 3. A Socio-demographic information questionnaire**

We would appreciate if you could fill out this questionnaire within 10 minutes. The information you provide is completely confidential and will be used only for academic research. Therefore, your participation and truthful answers based on your own condition are important to us. Thank you for your cooperation.

| **NO.** | **Questions-Stems** | **Options/Answers** |
| --- | --- | --- |
| C.1. | Gender | ☐Male ☐Female |
| C.2. | Age (years) |  |
| C.3. | What is the highest education level that you have obtained? | ☐Bachelor’s degree  ☐College degree  ☐Senior high school diploma  ☐Secondary school diploma  ☐Junior high school diploma |
| C.4. | How many years have you worked in the medical field? |  |
| C.5. | Have you ever dealt with snakebite? | ☐Yes ☐No |
|  |  |  |

**Part 4. Acceptance questionnaire of the SOP and checklist**

Group: ☐B ☐C

You will see a scale beside each item. This scale is numbered 1 to 5. Please put an “✔”in Table A4 next to the answer which you feel is best to describe each item. In addition, if you have other advice, please write that down at the bottom of Table A4. Thank you for your cooperation.

| **No.** | **Items** | **Strongly disagree** | **Disagree** | **Not sure** | **Agree** | **Strongly agree** |
| --- | --- | --- | --- | --- | --- | --- |
| D.1. | Met my learning needs | 1 | 2 | 3 | 4 | 5 |
| D.2. | Effectively updated my concept of treatment | 1 | 2 | 3 | 4 | 5 |
| D.3. | Stimulated my interest in learning | 1 | 2 | 3 | 4 | 5 |
| D.4. | Integrated theory with practice | 1 | 2 | 3 | 4 | 5 |
| D.5. | Easy to master | 1 | 2 | 3 | 4 | 5 |
| D.6. | Guided me to effective treatment | 1 | 2 | 3 | 4 | 5 |
| D.7. | Improved my self-confidence on treatment | 1 | 2 | 3 | 4 | 5 |
| D.8. | Enhanced decision-making ability | 1 | 2 | 3 | 4 | 5 |
| D.9. | Assisted me in enhancing my sense of self-identity | 1 | 2 | 3 | 4 | 5 |

Other advice:

|  |
| --- |

|  |
| --- |

|  |
| --- |

|  |
| --- |
